# Mitral Valve Surgery in Patients With Rheumatic Heart Disease: Repair vs. Replacement

**DOI:** 10.3389/fcvm.2021.685746

**Published:** 2021-05-28

**Authors:** Guangguo Fu, Zhuoming Zhou, Suiqing Huang, Guangxian Chen, Mengya Liang, Lin Huang, Zhongkai Wu

**Affiliations:** ^1^Department of Cardiac Surgery, First Affiliated Hospital of Sun Yat-sen University, Guangzhou, China; ^2^NHC Key Laboratory of Assisted Circulation, Sun Yat-sen University, Guangzhou, China

**Keywords:** mitral valve, repair, replacement, rheumatic heart disease, meta-analysis

## Abstract

**Background:** High morbidity and mortality caused by rheumatic heart disease (RHD) are global burdens, especially in low-income and developing countries. Whether mitral valve repair (MVP) benefits RHD patients remains controversial. Thus, we performed a meta-analysis to compare the perioperative and long-term outcomes of MVP and mitral valve replacement (MVR) in RHD patients.

**Methods and Results:** A systematic literature search was conducted in major databases, including Embase, PubMed, and the Cochrane Library, until 17 December 2020. Studies comparing MVP and MVR in RHD patients were retained. Outcomes included early mortality, long-term survival, freedom from reoperation, postoperative infective endocarditis, thromboembolic events, hemorrhagic events, and freedom from valve-related adverse events. Eleven studies that met the inclusion criteria were included. Of a total of 5,654 patients, 1,951 underwent MVP, and 3,703 underwent MVR. Patients who undergo MVP can benefit from a higher long-term survival rate (HR 0.72; 95% CI, 0.55–0.95; *P* = 0.020; *I*^2^ = 44%), a lower risk of early mortality (RR 0.62; 95% CI, 0.38–1.01; *P* = 0.060; *I*^2^ = 42%), and the composite outcomes of valve-related adverse events (HR 0.60; 95% CI, 0.38–0.94; *P* = 0.030; *I*^2^ = 25%). However, a higher risk of reoperation was observed in the MVP group (HR 2.60; 95% CI, 1.89–3.57; *P*<0.001; *I*^2^ = 4%). Patients who underwent concomitant aortic valve replacement (AVR) in the two groups had comparable long-term survival rates, although the trend still favored MVP.

**Conclusions:** For RHD patients, MVP can reduce early mortality, and improve long-term survival and freedom from valve-related adverse events. However, MVP was associated with a higher risk of reoperation.

**Systematic Review Registration**: https://www.crd.york.ac.uk/PROSPERO/display_record.php?RecordID=228307.

## Introduction

Currently, more than 30 million patients worldwide suffer from rheumatic heart disease (RHD), which leads to approximately 300,000 deaths and 10 million disabilities every year (1). High morbidity and mortality caused by RHD are global burdens, especially in low-income and developing countries (1–3). As RHD progresses, it can cause severe mitral stenosis (MS) and/or mitral regurgitation (MR) (4) and concomitantly affects more than 30% of patients' aortic valves (5). Although the current American College of Cardiology/American Heart Association guidelines prioritize the use of percutaneous mitral balloon commissurotomy (PMBC) for the intervention of RHD-related conditions (6), the use of this safe and effective method is restricted by the absence of left atrium thrombus and calcification and the severity of MR (7). Patients require surgical treatment when they reach New York Heart Association functional class III/IV, with surgical indications including severe calcification, concomitant valve/coronary disease, or previous unsuccessful PMBC (6, 7). Mitral valve replacement (MVR) and mitral valve repair (MVP) are the most widely used and effective surgical methods. The standard MVP techniques included commissurotomy, subvalvular debridement, ring annuloplasty, artificial chordae replacement, and release of subvalvular apparatus. Mitral valve repair (MVP) often requires different techniques according to the specific pathological features of the valves in different patients. Because MVP employs a higher-level surgical techniques than MVR and is based on a personalized surgical protocol and a higher level of professional knowledge, the MVP rate is limited by the surgeon's and institutional experience (8). The application of MVP in RHD is technically more difficult than that of MVR since lesions of the valve and subvalvular apparatus in RHD are severer than those in non-RHD disease (9, 10). Although MVP is recommended over MVR for degenerative mitral valve disease (11–13), whether MVP benefits patients with RHD remains controversial, and current guidelines do not provide clear recommendations. Therefore, we conducted a meta-analysis based current studies, with the aim of comparing the perioperative and long-term outcomes of MVP and MVR in patients with RHD.

## Materials and Methods

### Search Strategy

This meta-analysis was performed based on the Preferred Reporting Items for Systematic reviews and Meta-Analyses (PRISMA) guidelines and has been registered at the International Prospective Register of Systematic Reviews (PROSPERO), number CRD42021228307. A systematic literature search was conducted in major databases, including Embase, PubMed, and the Cochrane Library, until 17 December 2020. The following key terms were used: [RHD OR rheumatic OR (rheumatic heart disease) OR (rheumatic heart diseases) OR (Bouillaud's disease) OR (Bouillauds disease) OR (Bouillaud disease)] AND [annuloplasty OR annuloplasties OR (annulus repair) OR repair OR replacement] AND [mitral OR (mitral valve) OR (mitral valve surgery) OR (mitral valve reconstruction)]. Reference lists of relevant studies were also browsed to reduce possible omissions.

### Selection Criteria and Quality Assessment

The study inclusion criteria were as follows: (1) studies comparing MVP and MVR for patients with RHD; (2) patients over 15 years old; and (3) reporting of at least one of the following outcomes: early mortality, long-term survival, reoperation, postoperative infective endocarditis (IE), thromboembolic events, hemorrhagic events, and Kaplan-Meier curves of long-term survival or all-cause mortality. The exclusion criteria were as follows: (1) studies not published in English and (2) reviews, case reports, conference abstracts, and letters. When two studies came from the same institution and had overlapping populations, only the latest study was retained. All studies were screened independently by two authors (GF and ZZ), and all differences were resolved through discussion. The quality of each included study was evaluated and scored using the Newcastle-Ottawa Scale (NOS) checklist.

### Definition of Outcomes

The outcomes were defined as follows. Early mortality was defined as 30-day mortality after surgery due to any cause. Long-term survival was used to describe the survival time from discharge to death from any cause. Freedom from reoperation was considered freedom from reoperation involving the mitral valve. Valve-related adverse events included infective endocarditis, thromboembolic events (cerebral infarction, peripheral embolism, valve thrombosis, and transient ischemic attack) and hemorrhagic events (any serious bleeding event that resulted in death, hospitalization, permanent injury, or required blood transfusion).

### Data Extraction

The following data were extracted: lead author, publication year, country, study period, number of participants, follow-up years, study design, age, sex, diabetes mellitus, atrial fibrillation, MR, MS, MR+MS, concomitant aortic valve replacement (AVR), concomitant tricuspid valve repair (TVP), and the outcomes mentioned in the inclusion criteria. All data extraction was completed by two authors (GF and ZZ). Calibration was carried out after extraction, and any disagreements between the two authors were resolved by discussion or by seeking the opinion of another author (SH) until a consensus was reached.

### Statistical Analysis

Statistical analysis was performed by RevMan (version 5.4.1). A random-effects model with Mantel-Haenszel weighting was used to estimate the overall risk ratio (RR) and 95% confidence intervals (CIs) for variables with dichotomous outcomes, including early mortality, IE, thromboembolic events, and hemorrhagic events. For studies that did not directly report adjusted hazard risks (HRs), we obtained data from digitized Kaplan-Meier curves produced by Engauge Digitizer software (version 12.1) and then estimated the HRs according to the method introduced by Tierney et al. (14). Similarly, a random-effects model with Inverse-Variance weighting was used to calculate the overall HR and 95% CIs for long-term survival, freedom from reoperation and freedom from valve-related adverse events. Heterogeneity was investigated by the chi^2^ test and quantified by the *I*^2^ statistic. The heterogeneity and overall RR/HR described above are presented as forest plots. Additionally, we reconstructed the aggregated Kaplan-Meier curves by time, survival rate, and number at risk using the MetaSurv package from R software (version 3.6.3). Publication bias is shown by a funnel plot and was assessed statistically by Begg's test and Egger's test in Stata (version SE 15.1). Sensitivity analysis was conducted by excluding the studies with the largest proportions or those most likely to be biased and then reanalyzing the data. Subgroup analysis was performed based on different study designs, including propensity score matching (PSM) or unmatched studies, and whether concomitant AVR was performed in all patients.

## Results

### Study Characteristics

Through preliminary searches, a total of 1,159 relevant records were collected, of which 11 studies (13, 15–24) that met the inclusion criteria were included in our meta-analysis (Supplementary Figure 1). Among the eleven studies, four were based on PSM analysis, and seven were unmatched (Table 1). In addition, three studies focused on patients who had undergone mitral valve surgery with concomitant AVR (Table 2). In total, 5,654 patients were included, including 1,951 who underwent MVP and 3,703 who underwent MVR. The baseline characteristics of the included patients are presented in Table 2, and the quality assessment scores are presented in Supplementary Table 2.

**Table 1:** Main characteristics of the included studies.

**Studies**	**Patients MVP/MVR (*n*)**	**Country**	**Study period**	**Mean follow-up years**	**Study design**	**NOS scores**
Brescia 2020	80/100	USA	1997.9–2018.3	5.0 ± 3.9	Unmatched	8
Chen 2020	467/467	China (Taiwan)	2000.1–2013.12	5.9 ± 4.2/5.8 ± 4.2	PSM	8
Fu 2020	529/529	China	2011.1–2019.4	median 4.12	PSM	8
Kim 2018	188/188	South Korea	1997.1–2015.12	10.9 ± 2.3	PSM	8
Russell 2017	119/1078	Australia	2001.6–2013.12	NA	Unmatched	7
Geldenhuys 2012	69/69	South Africa	2000.1–2010.12	4.4 ± 3.0	PSM	9
Wang 2008	33/59	China (Taiwan)	1997.11–2005.7	2.8 ± 2.1/3.1 ± 1.8	Unmatched	7
Kuwaki 2007	47/81	Japan	1981–2003	9.1 ± 4.2/9.1 ± 4.6	Unmatched	7
Talwar 2007	76/293	India	1995.1–2005.12	5.8 ± 3.4/4.3 ± 3.1	Unmatched	8
Ho 2004	201/408	Vietnam	1992–2001	9.0	Unmatched	7
Yau 2000	142/431	Canada	1978.10–1995.6	5.7 ± 3.8	Unmatched	8

*MVP, mitral valve repair; MVR, mitral valve replacement; NA, not available; NOS, Newcastle-Ottawa Scale; PSM, propensity score matching (13, 15–24)*.

**Table 2:** Baseline characteristics of patients.

**Studies**	**Patients (*****n*****)**		**Age (years)**		**Female (%)**		**DM (%)**		**MR (%)**		**MS (%)**		**MR+MS (%)**		**AF (%)**		**CS AVR (%)**		**CS TVP (%)**	
	**MVP**	**MVR**	**MVP**	**MVR**	**MVP**	**MVR**	**MVP**	**MVR**	**MVP**	**MVR**	**MVP**	**MVR**	**MVP**	**MVR**	**MVP**	**MVR**	**MVP**	**MVR**	**MVP**	**MVR**
Brescia 2020	80	100	NA	NA	NA	NA	NA	NA	NA	NA	NA	NA	NA	NA	NA	NA	NA	NA	NA	NA
Chen 2020	467	467	56.8 ± 14.2	56.7 ± 13.8	53.1	55.9	14.6	16.5	19.3	18.8	65.1	65.5	15.6	15.6	57.4	58.0	22.1	21.0	26.3	27.2
Fu 2020	529	529	54.5 ± 10.9	54.6 ± 10.0	72.2	72.2	10.0	13.0	12.7	10.2	10.4	11.9	76.9	77.9	67.9	69.6	18.9	19.8	91.1	89.2
Kim 2018	188	188	48.8 ± 3.4	47.6 ± 2.1	71.8	75.5	11.7	13.8	50.5	47.3	30.3	33.0	19.1	19.7	64.9	63.8	23.4	20.2	38.3	35.6
Russell 2017	119	1078	57.3	62.0	58.0	73.1	14.3	21.1	81.5	63.9	25.2	75.3	NA	NA	26.1	48.9	NA	NA	NA	NA
Geldenhuys 2012	69	69	36.9 ± 14.5	40.9 ± 11.7	78.3	84.1	NA	NA	39.0	13.0	3.0	7.0	27.0	49.0	29.0	39.0	NA	NA	6.0	13.0
Wang 2008	33	59	49.7 ± 13.2	58.1 ± 11.2	63.7	66.1	12.1	6.8	NA	NA	NA	NA	NA	NA	93.9	96.6	NA	NA	45.5	61.0
Kuwaki 2007	47	81	48.0 ± 10.0	53.0 ± 8.0	70.2	58.0	2.1	7.4	8.5	6.2	83.0	54.3	8.5	39.5	NA	NA	100	100	17.0	40.7
Talwar 2007	76	293	30.3 ± 10.4	32.5 ± 10.7	30.3	27.8	NA	NA	15.8	13.2	40.8	44.4	43.4	42.4	48.7	38.3	100	100	NA	NA
Ho 2004	201	408	32.2 ± 10.4	38.7 ± 8.6	46.3	44.4	NA	NA	37.4	12.7	30.3	59.1	32.3	28.2	36.8	60.3	100	100	28.9	32.4
Yau 2000	142	431	42.0 ± 13.1	57.9	85.0	79.6	NA	NA	16.2	14.8	67.6	48.0	16.2	37.1	NA	NA	NA	NA	7.8	17.6

*MVP, mitral valve repair; MVR, mitral valve replacement; NA, not available; DM, diabetes mellitus; MR, mitral regurgitation; MS, mitral stenosis; AF, atrial fibrillation; CS, concomitant surgery; AVR, aortic valve replacement; TVP, tricuspid valve repair (13, 15–24)*.

### Early Mortality

**Figure 1:**
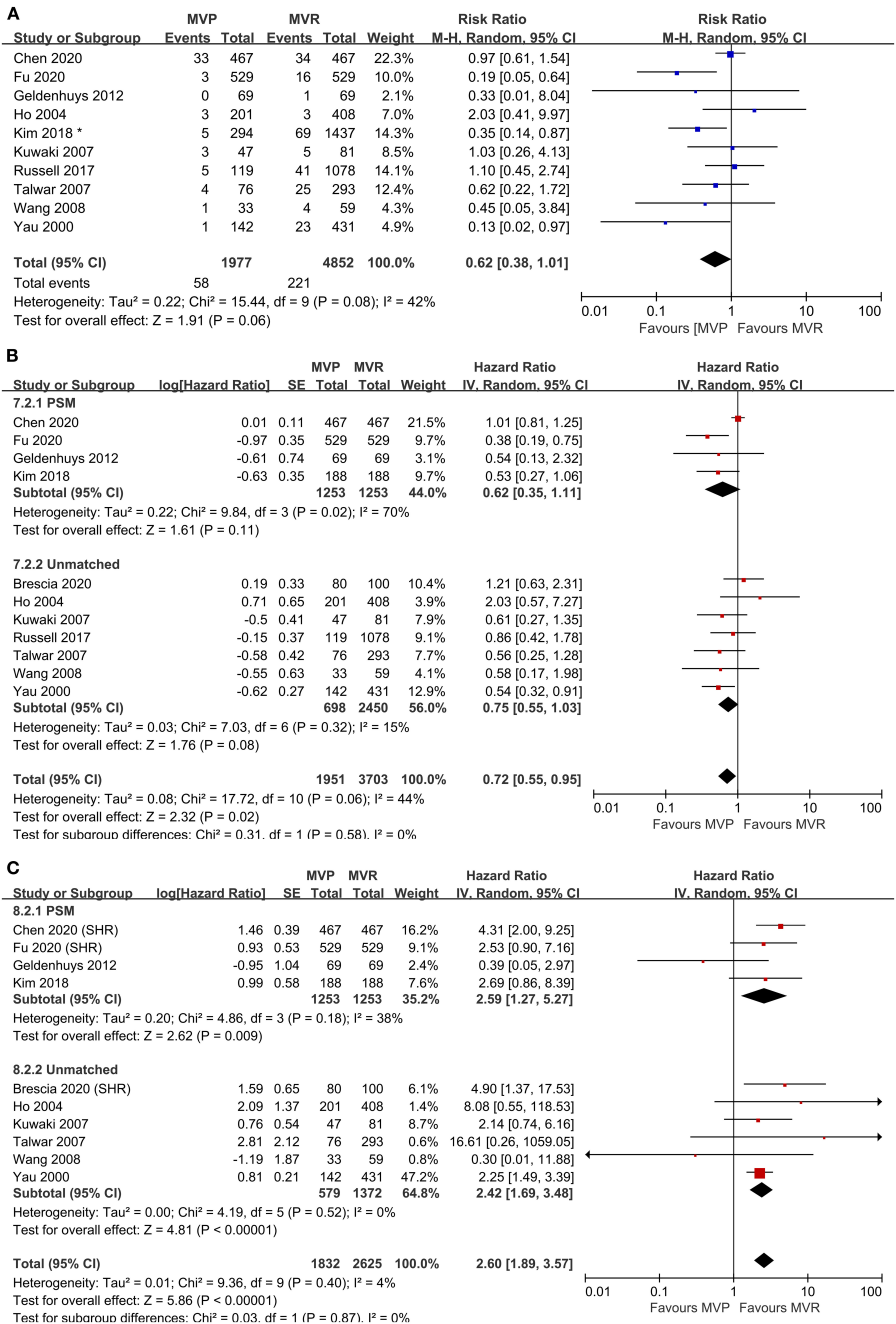
Forest plots showing the results of early and late outcomes. **(A)** Early mortality, **(B)** long-term survival, analyzed by study design, and **(C)** freedom from reoperation, analyzed by study design. MVP, mitral valve repair; MVR, mitral valve replacement; M-H, Mantel-Haenszel; CI, confidence interval; SE, standard error; IV, Inverse-Variance; PSM, propensity score matching; SHR, subdistribution hazard ratio; *before matching.

Ten studies were included in the analysis of early mortality (6,829 patients, 1,977 underwent MVP and 4,852 underwent MVR). The results indicated a trend in favor of MVP (RR 0.62; 95% CI, 0.38–1.01; *P* = 0.060; *I*^2^ = 42%) (Figure 1A), although no significant difference between the two groups was found. Mild heterogeneity was observed.

### Long-Term Survival

**Figure 2:**
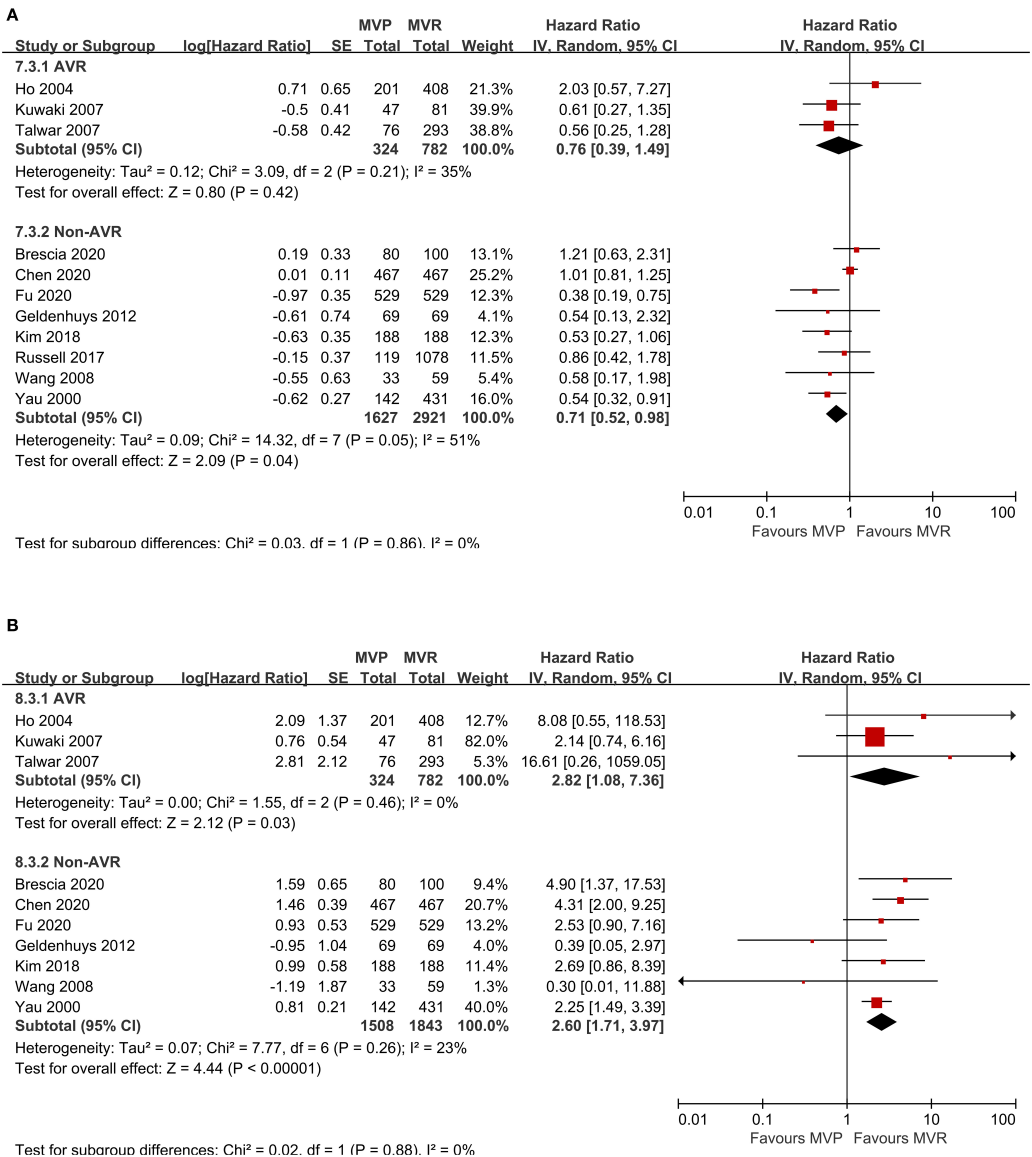
Forest plots showing the results of subgroup analysis according to whether concomitant AVR was performed. **(A)** Long-term survival and **(B)** freedom from reoperation. MVP, mitral valve repair; MVR, mitral valve replacement; AVR, aortic valve replacement; IV, Inverse-Variance; CI, confidence interval; SE, standard error.

A total of eleven studies were included (5,654 patients, 1,951 underwent MVP and 3,703 underwent MVR), of which four directly provided adjusted HRs; for the others, the HR-value and 95% CI were estimated from Kaplan-Meier curves. The analysis of these studies found that the MVP group had a significantly higher long-term survival rate than the MVR group (HR 0.72; 95% CI, 0.55–0.95; *P* = 0.020; *I*^2^ = 44%) (Figure 1B). However, a significant difference was not found in PSM studies (HR 0.62; 95% CI, 0.35–1.11; *P* = 0.110; *I*^2^ = 70%) or unmatched studies (HR 0.75; 95% CI, 0.55–1.03; *P* = 0.080; *I*^2^ = 15%). In addition, a significant difference was observed in the non-AVR group (HR 0.71; 95% CI, 0.52–0.98; *P* = 0.040; *I*^2^ = 51%), but not in the AVR group (HR 0.76; 95% CI, 0.39–1.49; *P* = 0.42; *I*^2^ = 35%) (Figure 2A). There was moderate heterogeneity in the PSM studies and the non-AVR group and mild heterogeneity in the overall result and the AVR group. Reconstructed Kaplan-Meier curves derived from 10 studies showed that the long-term survival rates at 4, 8, and 12 years were 90.11, 82.95, and 75.73%, respectively, in the MVP group and 88.78, 82.53, and 72.45%, respectively, in the MVR group (**Figure 4A**).

### Freedom From Reoperation

Ten studies were included (4,457 patients, 1,832 underwent MVP and 2,625 underwent MVR), of which four directly provided adjusted HRs or subdistribution hazard ratio (SHRs); in the other six, HRs and 95% CIs were estimated from Kaplan-Meier curves. The analysis of these studies indicated that the MVP group had a significantly higher reoperation rate than the MVR group (HR 2.60; 95% CI, 1.89–3.57; *P* < 0.001; *I*^2^ = 4%) (Figure 1C). A higher rate in the MVP group was also observed in the subgroups of PSM studies (HR 2.59; 95% CI, 1.27–5.27; *P* = 0.009; *I*^2^ = 38%), unmatched studies (HR 2.42; 95% CI, 1.69–3.48; *P* < 0.001; *I*^2^ = 0%), the AVR group (HR 2.82; 95% CI, 1.08–7.36; *P* = 0.030; *I*^2^ = 0%), and the non-AVR group (HR 2.60; 95% CI, 1.71–3.97; *P* < 0.001; *I*^2^ = 23%) (Figure 2B). There was mild heterogeneity in the PSM studies. Reconstructed Kaplan-Meier curves derived from 10 studies showed that the freedom from reoperation rates at 4, 8, and 12 years were 94.27, 87.87, and 74.45%, respectively, in the MVP group and 96.94, 92.74, and 86.66%, respectively, in the MVR group (**Figure 4B**).

### Freedom From Valve-Related Adverse Events

**Figure 3:**
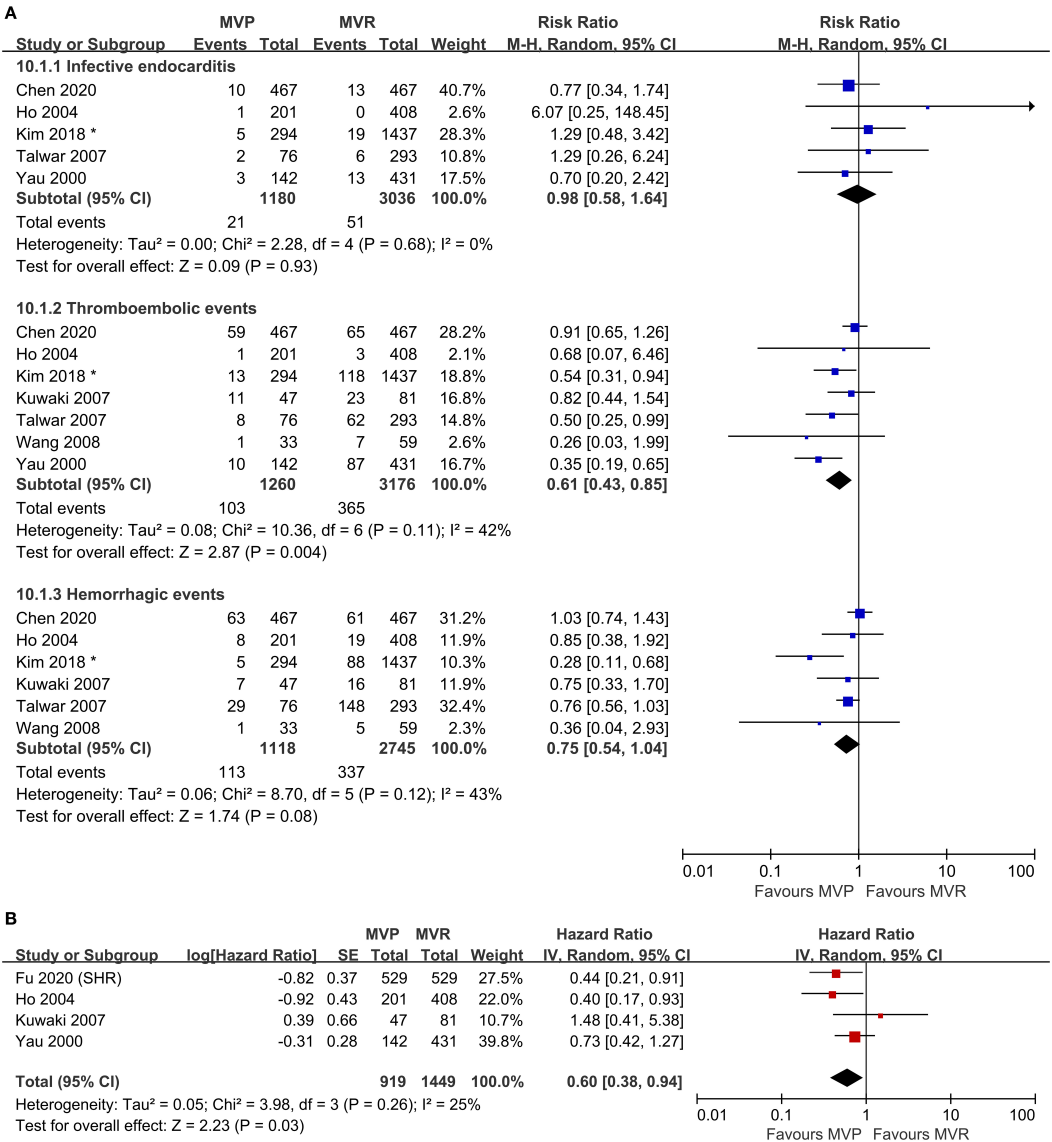
Forest plots showing the results for valve-related adverse events. **(A)** Adverse events, including infective endocarditis, thromboembolic events, and hemorrhagic events and **(B)** freedom from valve-related adverse events. MVP, mitral valve repair; MVR, mitral valve replacement; M-H, Mantel-Haenszel; CI, confidence interval; SE, standard error; IV, Inverse-Variance; SHR, subdistribution hazard ratio; *before matching.

**Figure 4:**
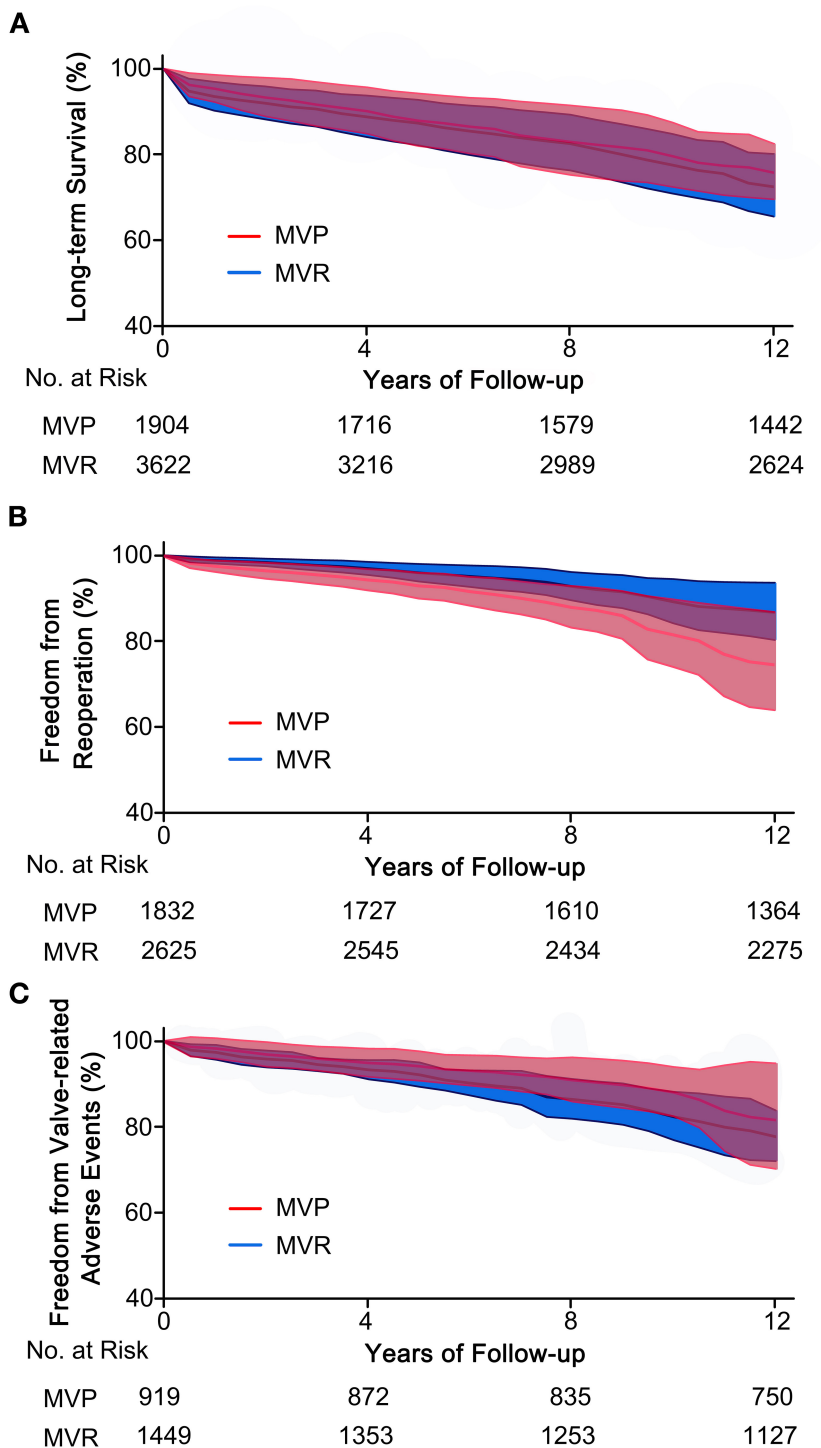
Reconstructed Kaplan-Meier curves for MVP and MVR in patients with RHD. **(A)** Reconstructed curves of long-term survival from 10 studies, **(B)** reconstructed curves of freedom from reoperation from 10 studies, and **(C)** reconstructed curves of freedom from valve-related adverse events from four studies. MVP, mitral valve repair; MVR, mitral valve replacement; RHD, rheumatic heart disease.

Five studies were included in the analysis of IE, seven studies were included in the analysis of thromboembolic events, and six studies were included in the analysis of hemorrhagic events. The risk of thromboembolic events in the MVP group was lower than that in the MVR group (RR 0.61; 95% CI, 0.43–0.85; *P* = 0.004; *I*^2^ = 42%), but the rates of IE (RR 0.98; 95% CI, 0.58–1.64; *P* = 0.93; *I*^2^ = 0%) and hemorrhagic events (RR 0.75; 95% CI, 0.54–1.04; *P* = 0.080; *I*^2^ = 43%) were not significantly different (Figure 3A). Four studies that reported the composite outcomes of valve-related adverse events also indicated a lower risk in the MVP group (HR 0.60; 95% CI, 0.38–0.94; *P* = 0.030; *I*^2^ = 25%) (Figure 3B). Reconstructed Kaplan-Meier curves derived from four studies showed that the freedom from valve-related adverse event rates at 4, 8, and 12 years were 94.91, 90.91, and 81.59%, respectively, in the MVP group and 93.36, 86.47, and 77.75%, respectively, in the MVR group (Figure 4C).

Neither Begg's test nor Egger's test indicated statistically significant publication bias in terms of early mortality, long-term survival, freedom from valve-related adverse events, IE, thromboembolic events, and hemorrhagic events. Funnel plots are provided in the supplemental materials (Supplementary Figure 2).

## Discussion

Our meta-analysis demonstrated that in RHD patients, MVP showed significant advantages over MVR in long-term survival rate and valve-related adverse events, especially thromboembolic events. However, the risk of mitral valve reoperation in the MVP group was significantly higher than that in the MVR group. No significant difference was found in IE and hemorrhagic events. However, for RHD patients with concomitant AVR, the long-term survival benefit of MVP was not obvious, and the reoperation rate of MVP was still higher than that of MVR.

Several published previously meta-analyses have compared MVP with MVR in RHD patients. In 2013, Wang et al. (25) included seven studies and reported that MVP provided longer short-term and long-term event-free survival for adult RHD patients. However, for unclear reasons, they included a study that contained young children (26), and the number of studies they included was small. Saurav et al. (27) believed that RHD patients with concomitant AVR were better suited for MVR than for MVP because of the lower rate of reoperation and the absence of a significant difference in long-term survival in their subgroup analysis. However, they did not take time to events into account and used RR rather than HR to assess the long-term outcomes in their analysis. Our meta-analysis included the latest comparative studies, reported time-to-event outcomes, and reconstructed intuitive Kaplan-Meier curves, which provides new evidence for surgical decision-making.

Regarding early mortality, our meta-analysis showed a trend favoring MVP, although it did not show that MVP had a significant reduction effect compared to MVR, which is not consistent with the conclusions of previous studies (25). The 30-day mortality and 1-year mortality after MVP have been shown to be associated with the cardiac surgeon's total annual surgical volume (28, 29). In the study by Chen et al. (15), the multicenter data they collected from the database included fewer than 500 patients with MVP over 14 years. We could estimate that the average annual operation volume per center was small. The low volume of annual operations reflected insufficient experience with repair surgery among the cardiac surgeons, which can cause an increase in early mortality. In addition, they did not report the heart function data of the surgical patients; there may have been heterogeneity of cardiac insufficiency, which could present a significant limitation. We conducted a sensitivity analysis and eliminated this study, which accounted for the largest proportion and had potential bias. The results of the sensitivity analysis were completely contrary to the original conclusion (RR 0.55; 95% CI, 0.31–0.96; *P* = 0.030; *I*^2^ = 34%). Thus, we tend to consider MVP better for early mortality.

In terms of the long-term survival rate, the analysis of all of the included studies showed that the long-term survival rate of MVP was higher, and this conclusion remained stable (HR 0.65; 95% CI, 0.50–0.86; *P* = 0.002; *I*^2^ = 15%) after the study with the highest proportion was removed. In the PSM subgroup analysis, the sensitivity analysis indicated that the high heterogeneity was caused by Chen et al. (15), and the conclusion became completely homogeneous and statistically significant once that study was removed (HR 0.46; 95% CI, 0.29–0.73; *P* < 0.001; *I*^2^ = 0%). For patients who have only undergone mitral valve surgery, the benefits of MVP are well-explained. On the one hand, MVP can better preserve the subvalvular apparatus to protect the normal physiological function of the left ventricle (30). Tirone et al. (31) believed that even mild impairment of left ventricle function could adversely affect long-term survival. As a result, deaths associated with left ventricular failure are reduced after MVP. On the other hand, MVP avoids the long-term use of anticoagulants after surgery, which effectively reduces coagulation-related complications (20, 25, 32). From the perspective of MVP development, the survival rate of RHD patients after mitral valve surgery has improved significantly over the past decade in conjunction with a greater understanding of MVP and more reasonable assessments of the feasibility of MVP (13). In addition to long-term mortality, MVP also decreased the total treatment cost during the survival period of over 10 years (33). Patients who underwent MVP did not have to bear the burden of prosthetic valves and ongoing anticoagulant use. This additional benefit of MVP is undoubtedly more cost-effective for developing countries with the ability to implement MVP. However, although the trend was in favor of the AVR+MVP group in the subgroup analysis, RHD patients in this group did not appear to survive longer. Therefore, it can be considered that the long-term survival rate of patients who underwent AVR+MVP or AVR+MVR may not be substantially different. The use of AVR made long-term anticoagulation necessary, thus increasing complications, which may reduce the long-term survival benefits of MVP for patients (21). Currently, few cohort studies have compared these two groups, and conclusions should be drawn with caution. Similarly, AVR+MVP reduced hospitalization costs compared to AVR+MVR (34).

Without exception, the overall and subgroup analysis results for reoperation indicatedthat the MVP group was more likely to undergo mitral valve surgery again. The inevitable residue of diseased valve tissue, the presence of lesions involving the subvalvular apparatus, and younger age may make the reoperation rate significantly higher with MVP than with MVR (27, 35). The continuous natural progression of RHD also inevitably reduces the durability of repair surgery. In degenerative valve disease, a high recurrence of MVP after surgery has also been found by Ciarka et al. (36). However, several studies (35, 37, 38) have demonstrated that valve durability after the repair of RHD was equivalent to that of degenerative valve disease in a long-term follow-up. Previous studies reported that the mortality rate (39, 40), the acute kidney injury rate, and the possibility of longer ICU and hospital stays (41) after the second MVR operation tended to be higher than those rates for MVP. It is known that MVP requires that surgeons have more surgical skills and professional capabilities. In addition to the early mortality mentioned above, repair durability was also related to the annual volume of surgery performed by cardiac surgeons (28). Zhou et al. (42) reported that surgery before atrial fibrillation and left ventricular dysfunction improved the durability of MVP. Therefore, early MVP performed by experienced surgeons was considered to reduce the risk of reoperation.

The incidence of valve-related adverse events, especially thromboembolic events, was lower for patients who underwent MVP in this study. We removed the study with the highest proportions of each item for a sensitivity analysis, and the conclusions remained unchanged. Hemorrhagic events are a common complication with long-term use of anticoagulant drugs. The results indicated that MVP had a favorable effect on hemorrhagic events, although we did not find a significant difference between the two groups. Mitral valve repair (MVP) was associated with a lower risk of thromboembolic events. Long-term oral anticoagulant use after MVR requires a narrow range of international normalized ratios (INRs), and excessively high or low INRs can cause complications (43, 44). Hemorrhagic and thromboembolic events were more likely in the MVR group as a result of decreased long-term drug compliance and the maintenance time of the INR standard range (45, 46). In another study (27), the benefits of MVP in terms of thromboembolic events were demonstrated to extend to RHD patients with concomitant AVR.

It should be pointed out that for children and young unmarried women, MVR is not the optimal surgical method. The degeneration of biologic prostheses is not suitable for growing children. Lifelong anticoagulation of mechanical prostheses has adverse consequences for growth in children and during pregnancy in young women (32). For patients who need MVR because of non-reparable valve structural lesions or unsuccessful repair, a retrospective cohort study showed that compared to biological prostheses, mechanical prostheses reduced the risk of death and reoperation, although it increased the possibility of bleeding (47). This benefit continued until the age of 70 years, while another study suggested that the age threshold should be considered at 65 (48). For people over 70 years old, the risk of hemorrhagic and thromboembolic events caused by biological prostheses was lower, associated with a better survival advantage (47).

## Limitation

We must acknowledge that our meta-analysis has some limitations. First, the included studies were mainly retrospective cohort studies, and patients were not randomly divided into MVP and MVR groups. Usually, the surgical treatment of a patient is primarily decided at the surgeon's discretion and inevitably leads to selection bias. Second, these studies came from different centers. There may be heterogeneity due to variations in surgical techniques and patient baseline conditions, such as the proportion of concomitant coronary artery bypass grafting, the NYHA functional class, and the mean left ventricular ejection fraction. Third, the data extracted from the Kaplan-Meier curves before merging was obtained through digital estimation. The digitalization and aggregation of survival curves inevitably reduced the accuracy of the primary data and led to potential deviation. Forth, few included studies have reported the detailed information of subvalvular apparatus. More well-designed studies are warranted to further explore the function of subvalvular apparatus in mitral valves and its clinical implications in mitral valve surgery. Finally, limited by the information provided by the included studies, no further stratification according to the use of biological prostheses vs. mechanical prostheses in MVR or the age of the patient can be performed.

## Conclusion

For RHD patients, MVP can reduce early mortality, and improve long-term survival and freedom from valve-related adverse events, although it was associated with a higher risk of reoperation. The results remained generally consistent in patients undergoing concomitant AVR. In summary, MVP is a promising strategy for selective RHD patients, and experienced cardiac centers are encouraged to apply MVP more routinely in clinical practice.

## Data Availability Statement

The raw data supporting the conclusions of this article will be made available by the authors, without undue reservation.

## Author Contributions

GC and ML: conception and design. ZW: administrative support. GF, ZZ, and SH: literature search, selection and quality assessment, and data extraction. GF and ZZ: data analysis and interpretation. All authors contributed to the article and approved the submitted version.

## Conflict of Interest

The authors declare that the research was conducted in the absence of any commercial or financial relationships that could be construed as a potential conflict of interest.
