# Supplementary material for: Mitral Valve Surgery in Patients With Rheumatic Heart Disease: Repair vs. Replacement
Source: Front Cardiovasc Med. 2021 May 28;8:685746. doi: 10.3389/fcvm.2021.685746 (PMC8193043; doi:10.3389/fcvm.2021.685746)
Supplement: Supplementary file 1 [file Data_Sheet_1.pdf]

Figures and Figure legends

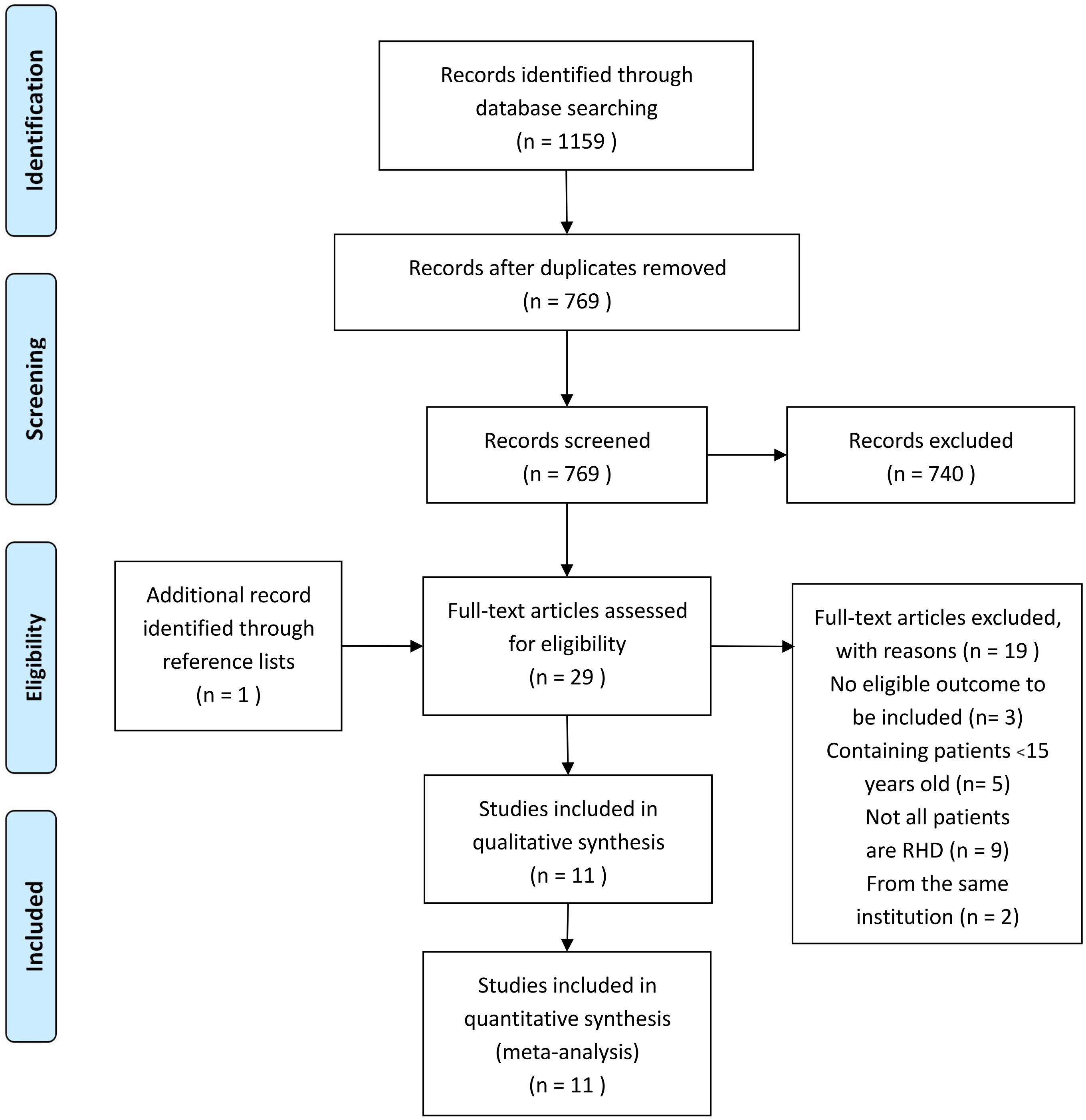

Supplementary Figure S1. PRISMA flow diagram for literature search and inclusion

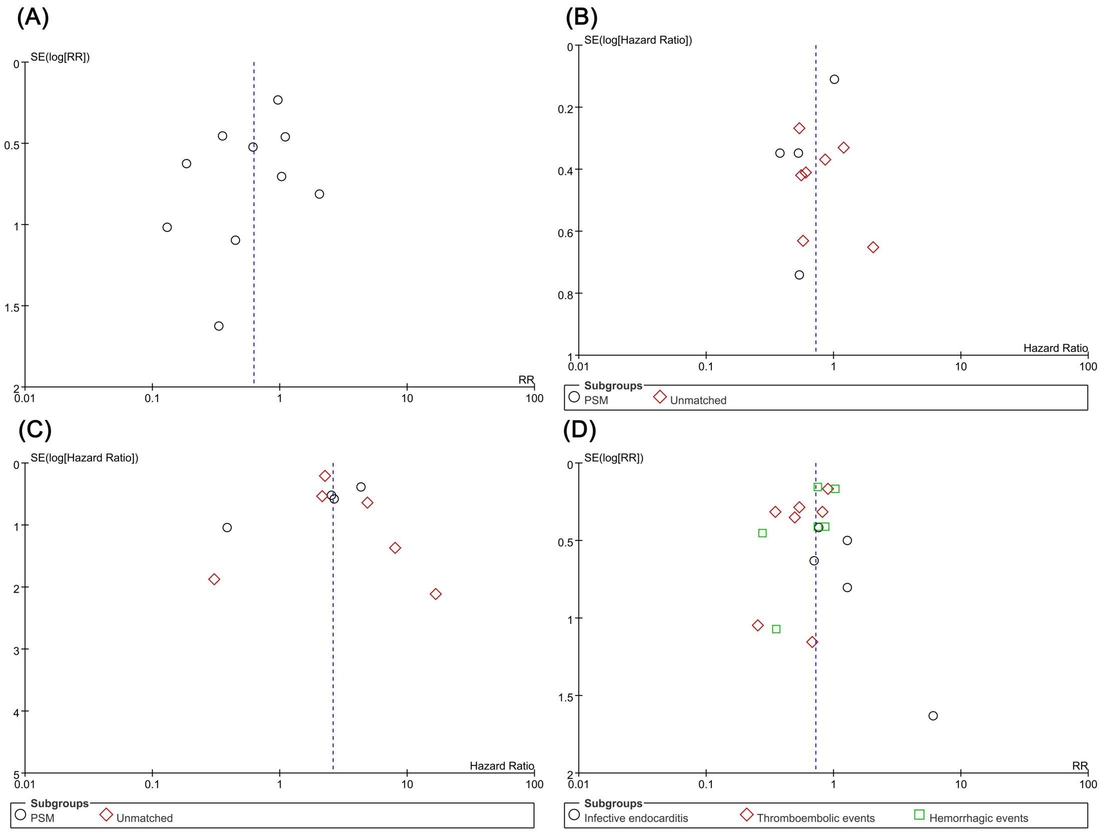

Supplementary Figure S2. Funnel plot showing the publication bias. A, Early mortality; B, long-term survival; C, freedom from reoperation; D, infective endocarditis, thromboembolic events and hemorrhagic events.

Table

Supplementary Table S1. Quality assessment of included studies using Newcastle–Ottawa Scale

	Selection					Outcome			
	Exposed cohort	Nonexposed cohort	Ascertainment of exposure	Outcome of interest	Comparability	Assessment of outcome	Length of follow-up	Adequacy follow-up	Total score
Brescia 2020	√	√	√	√	√	√	√	√	8
Chen 2020	√	√	√	√	√ √	√	√		8
Fu 2020	√	√	√	√	√ √	√	√		8
Kim 2018	√	√	√	√	√ √	√	√		8
Russell 2017	√	√	√	√	√	√	√		7
Geldenhuys 2012	√	√	√	√	√ √	√	√	√	9
Wang 2008	√	√	√	√	√	√	√		7
Kuwaki 2007	√	√	√	√	√	√	√		7
Talwar 2007	√	√	√	√	√	√	√	√	8
Ho 2004	√	√	√	√	√	√	√		7
Yau 2000	√	√	√	√	√	√	√	√	8
